# Evaluation of Biological Plant Protection Products for Their Ability to Induce Olive Innate Immune Mechanisms and Control *Colletotrichum acutatum*, the Causal Agent of Olive Anthracnose

**DOI:** 10.3390/plants13060878

**Published:** 2024-03-19

**Authors:** Maria Varveri, Anastasia G. Papageorgiou, Dimitrios I. Tsitsigiannis

**Affiliations:** Laboratory of Plant Pathology, Department of Crop Science, Agricultural University of Athens, Iera Odos 75, 11855 Athens, Greece; varvmar@gmail.com (M.V.); anastasia.papageorgiou.g@gmail.com (A.G.P.)

**Keywords:** olive tree, olive anthracnose, *Colletotrichum acutatum*, biological control, plant protection products, plant defense genes, olive innate immune system

## Abstract

Olive anthracnose is the most important fungal disease of the olive fruit worldwide, with the fungus *Colletotrichum acutatum* as the main cause of the disease in Greece. A total of 11 commercial biological plant protection products (bioPPPs) (Amylo-X^®^, Botector^®^, FytoSave^®^, LBG 01F34^®^, Mevalone^®^, Polyversum^®^, Remedier^®^, Serenade^®^ ASO, Sonata^®^, Trianum-P^®^, Vacciplant^®^), with various modes of action against the fungus *C. acutatum*, were evaluated by bioassays using detached fruits of two important olive Greek varieties, *cv*. Koroneiki and *cv*. Kalamon. Subsequently, the most effective bioPPPs were evaluated for their ability to induce plant defense mechanisms, by determining the expression levels of ten *Olea europaea* defense genes (*Pal*, *CuaO*, *Aldh1*, *Bglu*, *Mpol*, *Lox*, *Phely*, *CHI-2*, *PR-10*, *PR-5*). Remedier^®^, Trianum-P^®^, Serenade^®^ ASO, Sonata^®^, and Mevalone^®^ were the most effective in reducing disease severity, and/or inhibiting the conidia production by the fungus at high rates. Post bioPPPs application, high expression levels of several olive plant defense genes were observed. This study provides insights into commercial bioPPPs’ effectiveness in controlling olive anthracnose, as well as biocontrol-agents-mediated modulation of olive defense mechanisms.

## 1. Introduction

Olive anthracnose is a major disease of olives in many countries [1], and it is thought to be the most important and devastating fungal disease of the olive trees worldwide [2,3]. When exposed to high environmental humidity, infected fruits develop a soft, dark brown rot. They also produce a significant amount of an orange gelatinous substance that contains conidia, which are spores emerging from fungal fruiting bodies called acervuli and are responsible for disseminating the pathogen. Conversely, in dry conditions, the infected fruits become dehydrated, causing them to mummify and experience weight loss [2]. Symptoms of the disease can also be present on tree twigs and leaves, resulting in necrotic areas or even the shedding of leaves [4,5]. Furthermore, the presence of latent *Colletotrichum* infections on olive flowers during their early stages is responsible for causing blossom blight [5,6,7]. Growing outbreaks of *C. acutatum sensu stricto*-caused olive anthracnose in olive-producing countries have been reported lately [8], which is primarily influenced by the altered climatic conditions and the susceptibility of different cultivars, as well as the olive drupes’ ripening stage [9].

Olive anthracnose can be caused by various species within the *Colletotrichum* genus [2,10,11]; however, the majority of fungal isolates associated with olive anthracnose are clustering to the *C. acutatum* species complex [11]. In Greece, in line with the research conducted by Kolainis et al. in 2020 [12] and Iliadi et al. [5], all isolates obtained from infected drupes in Southern Greece (Peloponnese) were identified as *C. acutatum*. This finding confirms that *C. acutatum sensu stricto* is the dominant species responsible for causing olive anthracnose in Greece [12]. Furthermore, unpublished data obtained by the authors reveal that the *Colletotrichum* species most commonly isolated from heavily infected olive-producing regions in Greece is *C. acutatum sensu stricto*. This conclusion was reached using multilocus sequence typing (MLST) analysis.

Controlling olive anthracnose is a challenging task due to its high dependence on climatic conditions (temperature, humidity) for its spread and growth [13]. In general, the methods employed to manage olive anthracnose involve integrated approaches that combine various means and tools. These methods aim to either prevent the disease indirectly or directly, protecting the olive tree from anthracnose [2,14], and mainly rely on the use of synthetic fungicides [15]. Nonetheless, the prolonged use of these fungicides has led to the emergence of reduced sensitivity to them. This is a result of the selective pressure exerted by the fungicides, which favors the growth of resistant strains and reduces the overall diversity within nonpathogenic species [16]. As a consequence, these pressures stimulate mechanisms of variation through genetic mutations caused during the asexual or sexual reproduction, which quickly diminishes the effectiveness of fungicides in real-world conditions [17].

The direct control methods and tools for managing olive anthracnose also include the application of natural products and biocontrol agents, which have been increasingly utilized in recent times [13]. A group of biocontrol agents that could potentially be used for the control of olive anthracnose is endophytic fungi. These fungi are microorganisms that colonize the internal tissues of the plant either partially or throughout their entire life cycle, without causing obvious harm to their host [18]. Specifically, the mechanisms by which these microorganisms protect the plant host mainly rely on secondary metabolites such as alkaloids, peptides, steroids, terpenoids, and other volatile organic compounds [19,20]. Most of these compound categories include plant hormones, phytotoxins, and antimicrobial molecules, as well as antibiotics that can directly reduce disease severity through antibiosis, mycoparasitism, and competition, and indirectly induce plant defense responses [21]. Additionally, endophytic fungi are known to produce degradative enzymes (e.g., chitinases, proteases, and glucanases) that have the ability to break down the cell walls of pathogens [22]. All the aforementioned mechanisms can potentially work cooperatively [13]. The genus *Trichoderma* is one of the most studied genera [20] and also one of the most promising genera in terms of controlling anthracnose caused by species belonging to *Colletotrichum*, as concluded from in vivo experiments in various crops and different species of the genus *Colletotrichum* [13]. An alternative approach to disease management involves the application of epiphytic and endophytic bacteria, which compete with the pathogens of the plant for resources and, thus, reduce the severity of the disease [23]. Studies have indicated some underlying causes, including competition for nutrients between the competing bacterium and the pathogen, and the production of substances that can be harmful to the pathogen [24]. Additionally, as mentioned earlier for endophytic fungi, biological control is achieved through mechanisms that can be classified as competition, parasitism, antibiosis, and induction of host defense, with all these mechanisms likely to act cooperatively in this competitive relationship [23,25]. *Bacillus amyloliquefaciens* (former subtilis) is among the most well-studied biocontrol agents for the control of plant pathogens and post-harvest diseases [26]. It has also been studied in agricultural conditions in recent years with curative use [26,27]. It has been repeatedly observed that bacteria of the genus *Bacillus* achieve significant reduction in the mycelial growth of anthracnose-related pathogens [28], which is attributed to the metabolites they produce [26]. Several studies have shown that the toxic metabolites produced by *B. subtilis* not only inhibit plant pathogenic fungi growth, but also prevent the germination of their spores [26]. Sahile and colleagues (2008) [29] showcased that the *Bacillus* species seem to be promising biological control agents (BCAs) due to their simple nutritional requirements that allow them to colonize dry surfaces for extended periods, and their endospore-resistant structures formed to withstand unfavorable environmental conditions. Furthermore, plant-growth-promoting rhizobacteria (PGPRs) can represent another useful group for disease management. Relevant experiments by Lamsal and colleagues (2012) [30] applying PGPRs against pepper anthracnose caused by the fungus *Colletotricum acutatum* demonstrated impressive results both in terms of plant growth and inhibition of fungal development in the presence of these bacteria. Plant extracts have also been evaluated for their potential to control olive anthracnose. Pomegranate extract has effectuated in reducing the disease than copper compounds under field conditions [31].

Plants have the ability to actively react to different environmental factors such as gravity, light, temperature, physical stress, water, and nutrient availability. Additionally, they can respond to various chemical signals produced by microbes present in the soil and on other plants. These signals can either trigger or prepare the plant’s defense mechanisms by causing biochemical changes that improve its resistance against future infections by different pathogens [32]. Induced resistance is a natural plant defense mechanism against a variety of pathogens, including bacteria, viruses, fungi, and oomycetes (IR) [33]. Various experiments have shown that secondary metabolites of *Bacillus subtilis* and *Bacillus amyloliquefaciens* have the ability to activate induced systemic resistance (ISR) and, at the same time, reduce the severity of diseases caused by pathogenic bacteria or fungi [34,35,36].

To date, only a limited number of studies have been carried out to investigate the biocontrol potential of bioPPPs against *Colletotrichum acutatum* in in vivo conditions (detached fruits, field settings, and/or greenhouses). Furthermore, even fewer studies have elucidated the mode of action of these biocontrol agents (BCAs), such as the suppression of the disease via their ability to induce olive innate immune mechanisms. The goal of this study was to evaluate the efficacy of a total of 11 commercial biological plant protection products (bioPPPs) on the control of *C. acutatum* on the two most important and widely grown Greek olive (*Olea europaea* L.) varieties and to subsequently investigate their potential to induce the expression of defense-related genes in *cv*. Kalamon olive plants. The study will help the growers to apply the most promising bioPPPs for the control of this major disease for table olives and olive oil production in Greece.

## 2. Results

### 2.1. In Situ Evaluation of Biological Plant Protection Products (PPPs) on cv. Koroneiki

**Figure 1:**
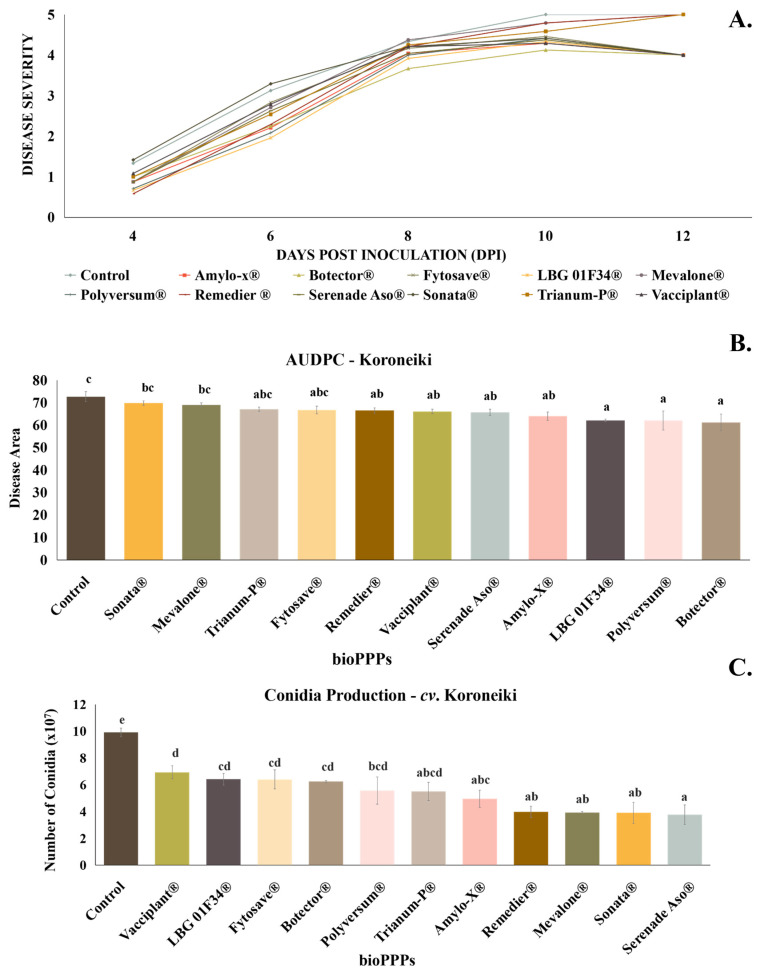
(**A**) Disease progress curve for bioPPPs–*cv*. Koroneiki. (**B**) AUDPC chart for biological plant protection products–*cv*. Koroneiki. (**C**) Number of conidia produced by *C. acutatum* for each treatment on *cv*. Koroneiki drupes. Different letters indicate statistical differences among treatments after one-way ANOVA followed by Tukey’s multiple comparison post hoc test (*p* < 0.05).

Eleven biological plant protection products (PPPs) were evaluated for their effectiveness in controlling the plant pathogenic fungus *C. acutatum* in olive drupes. During the disease assessment on *cv*. Koroneiki olive drupes, the first symptoms were observed on the fourth day (Figure 1A) post inoculation with the pathogen. Commercial bioPPPs demonstrated a particularly reduced effectiveness in diminishing the severity of the disease, as none of them led to a decrease of more than 16% (Figure 1B). The inhibition rates with reference to the positive control of the experiment were about 11.93% for Amylo-X^®^, 15.80% for Botector^®^, 8.09% for FytoSave^®^, 14.54% for LBG 01F34^®^, 5.02% for Mevalone^®^, 14.57% for Polyversum^®^, 8.49% for Remedier^®^, 9.61% for Serenade^®^ ASO, 3.93% for Sonata^®^, 2.81% for Trianum-P^®^, and 9% for Vacciplant^®^. Despite their inability to minimize disease severity, various biological fungicides showed a significant reduction concerning the production of conidia by the fungus *C. acutatum* on *cv*. Koroneiki fruits. More specifically, Amylo-X^®^, Mevalone^®^, Remedier^®^, Serenade^®^ ASO, Sonata^®^, and Trianum-P^®^ inhibited conidia production at rates that surpassed 40% with reference to the positive control (Figure 1C).

### 2.2. In Situ Evaluation of Plant Protection Products (PPPs) on cv. Kalamon

**Figure 2:**
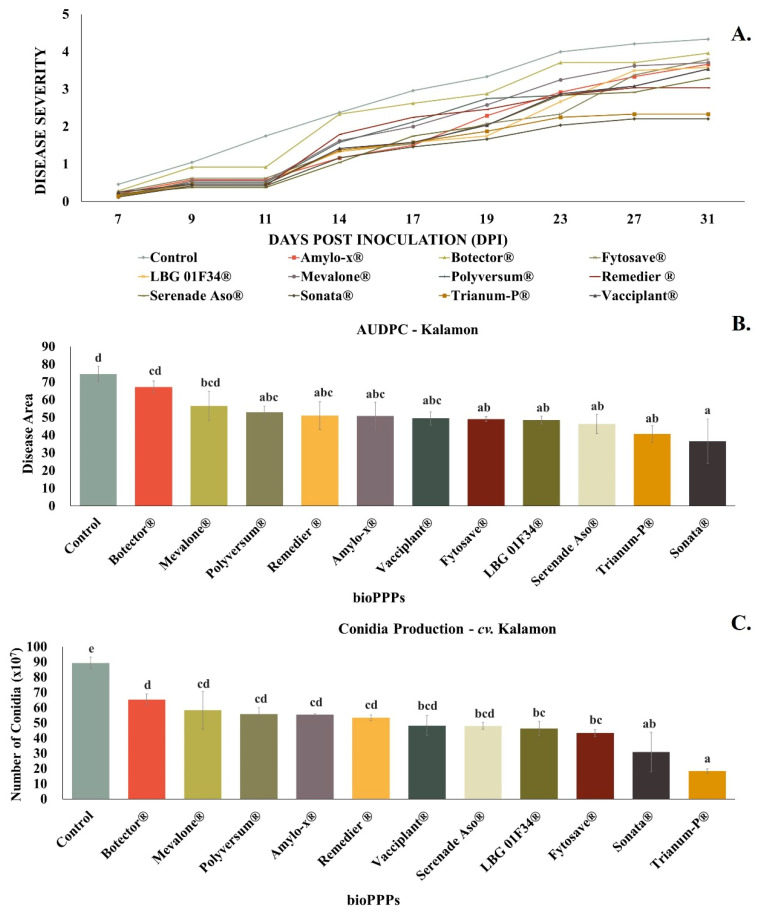
(**A**) Disease progress curve for bioPPPs–*cv*. Kalamon. (**B**) AUDPC chart for biological plant protection products–*cv*. Kalamon. (**C**) Number of conidia produced by *C. acutatum* for each treatment on *cv*. Kalamon. Different letters indicate statistical differences among treatments after one-way ANOVA followed by Tukey’s multiple comparison post hoc test (*p* < 0.05).

As depicted in Figure 2A, the observation of rot symptoms on *cv*. Kalamon fruits started at the seventh day post inoculation. BioPPPs demonstrated higher efficacy in reducing disease severity on *cv*. Kalamon, as 75% of the biological PPPs tested managed to reduce the disease at rates above 30–40% (Figure 2B), demonstrating a clear difference of the efficacy of bioPPPs depending on the used olive variety.

More specifically, the inhibition rates in comparison to the positive control of the experiment during the last observation (31 dpi) were about 31.78% for Amylo-X^®^, 9.81% for Botector^®^, 34.15% for FytoSave^®^, 34.88% for LBG 01F34^®^, 24.09% for Mevalone^®^, 29.04% for Polyversum^®^, 31.41% for Remedier^®^, 37.81% for Serenade^®^ ASO, 50.87% for Sonata^®^, 45.36% for Trianum-P^®^, and 33.62% for Vacciplant^®^.

Regarding conidia production, Trianum-P^®^ inhibited the produced conidia by *C. acutatum* with rates that exceeded 70%, and, in combination with the ability to minimize disease symptoms, was thought to be the most effective among the bioPPPs for *cv*. Kalamon. Nevertheless, Sonata^®^, FytoSave^®^, and LBG 01F34^®^ demonstrated significant reduction rates too (Figure 2C). Botector^®^ was thought to be the least effective, as it decreased neither the disease nor conidia production.

### 2.3. Quantification of Olive Innate Immunity Gene Expression Levels

**Figure 3:**
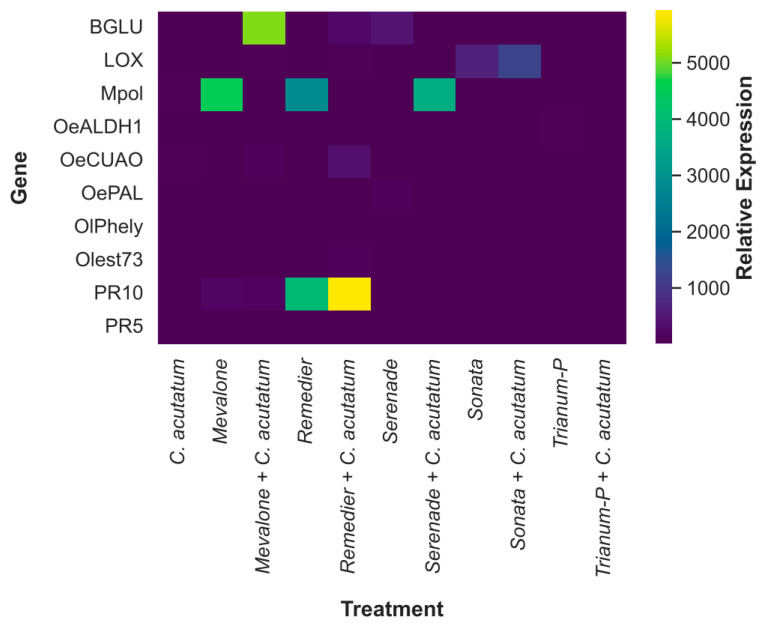
Expression patterns for the inoculated with *C. acutatum* olive plants, for plants post bioPPPs application and for plants post bioPPPs application and inoculation with the pathogen.

The expression levels of 10 genes known to be involved in the olive defense mechanisms were analyzed using the RT-qPCR method in plants treated with the five most effective biological plant protection products (Mevalone^®^, Sonata^®^, Serenade^®^ ASO, Remedier^®^, Trianum-P^®^) based on these studies. The experiments were carried out on two-year-old plants of *cv*. Kalamon. As depicted in Figure 3, post inoculation with the pathogen *C. acuatum*, none of the olive defense-related genes were significantly overexpressed when compared to the mock-treated plants of the experiment that had neither undergone any treatment nor inoculation with the pathogen. Mevalone^®^ led to a relative overexpression of the *PR10* gene in plants applicated either alone or before the inoculation with the fungus. Contrariwise, the *Mpol* gene showcased very high expression levels post Mevalone^®^ application, while a suppression of this gene was observed in plants where Mevalone^®^ was applied but, subsequently, an artificial inoculation with *C. acutatum* took place. On the contrary, *BGLU* demonstrated a high overexpression in plants where Mevalone^®^ and the pathogen were simultaneously present, while in Mevalone^®^-treated plants, we did not observe any relative expression of this gene in such levels (Figure 3). Apparently, Mevalone^®^ and *C. acutatum* interacted in a way that significantly affected the expression of *BGLU*.

PR10 was also overexpressed in high levels when Remedier^®^ was applied in olive plants; however, this overexpression reached its highest levels in plants where Remedier^®^ application was followed by inoculation with the pathogen, indicating a possible synergistic action between *C. acutatum* and the active ingredients of this bioPPP *Trichoderma asperellum* and *T. gamsii* towards this gene’s expression. Unlike *PR10*, *Mpol* presented its highest levels of expression post Remedier^®^ application, while in plants where Remedier^®^ and *C. acutatum* were coapplied, a suppression of this gene expression was observed, implying the possibility that Remedier’s^®^ effects on gene expression were overridden or counteracted by the presence of *C. acutatum*. *OeCUAO* and *BGLU* showed a relative overexpression in plants where Remedier’s^®^ *Trichoderma* spp. and *C. acutatum* coexisted, indicating a possible interaction between the aforementioned microorganisms towards these genes’ expression as well. Contrarily, Trianum-P^®^ did not result in any upregulation of the genes under investigation.

Serenade’s^®^ active ingredient *Bacillus amyloliquefaciens* strain QST 713 effectuated in a relative overexpression of *OePAL* and *BGLU*, but this expression was suppressed in plants where the pathogen was present too. In contrast, *Mpol* was expressed in significantly high levels in plants where Serenade^®^ was applied, and subsequently were artificially inoculated with the pathogen, revealing a possible interaction between *B. amyloliquefaciens* and *C. acutatum* towards this gene’s upregulation (Figure 3). Sonata’s^®^ active ingredient *Bacillus pumilus* strain QST 2808 application generated an overexpression of the *LOX* gene, which reached a slightly higher level in plants where Sonata^®^ was applied and then inoculated with the pathogen.

## 3. Discussion

The use of bioPPPs containing *Trichoderma* spp. fungi as biocontrol agents demonstrated promising results in decreasing disease severity in *cv.* Kalamon olive drupes. However, they did not exhibit the same level of effectiveness for *cv.* Koroneiki, indicating the dependence on the olive variety of the efficacy of bioPPPs. Nevertheless, the products Remedier^®^ and Trianum-P^®^ showcased a notable ability to inhibit spore production of *C. acutatum* on *cv.* Koroneiki fruit, with inhibition rates surpassing 40% in comparison to the control of the experiment. Additionally, in *cv.* Kalamon, Trianum-P^®^ inhibited spore production by rates over 70%, while Remedier^®^ demonstrated a much lower inhibition rate of around 20–30%. The mechanisms of action that characterize *Trichoderma* spp. fungi include competition with the pathogens for nutrients and space, antibiotic production, mycoparasitism, promotion of plant growth, reduction in tolerance to abiotic stress, and stimulation of host defense mechanisms against pathogens [21]. In results obtained from another study, *T. harzianum* strain T-39, the biocontrol agent of commercial product TRICHODEX^®^ was effectively used in two in planta experiments on strawberry plants, in roots and leaves, to control *Colletotrichum acutatum* [37]. The effectiveness of various strains of *T. harzianum* was later attributed, by Freeman and coauthors (2004) [38], either to their antibiotic activity or to their mycoparasitism towards *C. acutatum*. Additionally, numerous studies indicate that *T. harzianum*, active ingredient of Trianum-P^®^, grows faster than *C. acutatum* and, thus, restricts its growth through competition for space and nutrients [39,40]. Moreover, the changes in the structure of the mycelium textures of all *C. acutatum* isolates, as well as their growth in a dual-culture system with *T. harzianum*, were attributed, by Soufi and coauthors (2020) [40], to the toxic character of the secondary metabolites of the fungus. Moreover, it is known that fungi belonging to *Trichoderma* spp. produce a plethora of antibiotic compounds, such as trichodermins, trichodermol, trichotoxin, harzianum A, and harzianolide [41]. The aforementioned compounds were considered responsible for inhibiting the growth of most *Colletotrichum* isolates obtained from pear, apple, sour cherry, and tomato fruits in the study by Živković and coauthors (2010) [42]. BioPPPs Remedier^®^ and Trianum-P^®^ were applied sequentially to Kalamon young olive trees in order to investigate the expression levels of ten selected genes believed to be involved in olive tree defense. Among the ten genes examined, *PR10* stood out as the only gene whose expression levels increased after the application of both of the aforementioned biological formulations, compared to the expression levels in plants that had not undergone any intervention. It was also found that this gene was downregulated in plants that had only been artificially infected with the fungus *C. acutatum*. Interestingly, in plants treated with Remedier^®^ and Trianum-P^®^, *Mpol* and *Aldh1* genes were expressed at significantly higher levels than in the mock plants, which had neither undergone any treatment nor inoculation with the pathogen. The PR10-related protein is associated with pathogenesis (PR) and is a well-known group for the defense response system under (a)biotic stress conditions [43]. In some cases, the *PR10* gene shows homology with ribonucleases, and some members of this gene group do, indeed, have weak ribonuclease activity [44]. In the study by van Loon and van Strien (1999) [45], the PR10 protein family was considered to be associated with the salicylic acid signaling pathway, and could potentially move plastically within the plant during infection. Yet, based on findings by Rockenbach and colleagues (2018) [46], the resistance exhibited by apple leaves against the fungus *Colletotrichum fructicola* was entirely due to hypersensitivity reactions and the expression of *PR1* and *PR10* genes. As for the *Mpol* and *Aldh1* genes, there are reports of their induction by other compounds, such as the active substance laminarin [47], which also appeared to reduce the severity of disease caused by the fungus *Spilocaea oleaginea*. *Mpol* in olive plants encodes the PR protein that exhibits 1,3-β-glucanase activity, while *Aldh1* is mainly involved in the production of phenolic compounds, many of which are implicated in plant defense. Based on the above, the induction of the aforementioned genes involved in the defense of olive plants may be one of the factors contributing to the inhibition of *C. acutatum* fungus growth by the plant protection products Remedier^®^ and Trianum-P^®^. Despite the numerous properties and mechanisms of action of *Trichoderma* species against phytopathogenic fungi, few studies have revealed their impact on *C. acutatum* growth, and fewer have investigated the induction of olive defense mechanisms, as these studies often focus on other crops.

On the other hand, bacteria belonging to *Bacillus* spp. are widely used for the control of plant pathogenic fungi that affect economically important crops [42]. In the current study, the PPPs containing strains of *Bacillus* species Amylo-X^®^, Serenade^®^ ASO, and Sonata^®^ did not lead to any significant reduction in disease severity on *cv.* Koroneiki drupes. However, they effectuated in less conidia production by the pathogen at rates above 40% for Amylo-X^®^ and above 50% for Serenade^®^ ASO and Sonata^®^. Contrariwise, on *cv.* Kalamon drupes, disease severity reduced at rates between 30–50%, while the greatest reduction in conidia production was achieved post Sonata^®^ application, with an inhibition rate above 40%. The commercial product Serenade^®^ ASO is registered for the control of *C. acutatum* in olive cultivation in Greece as its effectiveness has been proven by numerous studies. One of these studies was conducted by Moreira and coauthors (2014) [23], who found that Serenade^®^ had the ability to control the disease caused by *C. acutatum* in both in vitro and in vivo conditions. This was attributed to the production of thermally stable metabolites, which completely inhibited the fungal growth of the isolates under examination. As a matter of fact, strains of *B. amyloliquefaciens* (former *B. subtilis*) are known for producing a range of antifungal compounds [23,48]. Specifically, *B. amyloliquefaciens* QST 713 strain, the biocontrol agent of Serenade^®^ ASO, is known for producing more than 30 different lipopeptides, which form the basis of its mode of action. This mode of action differs from other fungicides’ and, therefore, can provide prevention resistance development, which is commonly observed with fungicides that act on a single target [49]. There is a plethora of studies indicating that the stage of the plant pathogenic fungus growth at which bacteria of the genus *Bacillus* show the highest effectiveness is the conidial germination stage. This finding is consistent with the results of the present study, as the highest inhibition rates observed were related to the production of conidia by *C. acutatum*. In field experiments, *B. subtilis* strain QST 713 significantly reduced the incidence of anthracnose infections in olive fruit caused by *Colletotrichum acutatum* and *Colletotrichum gloeosporioides* fungi [50]. More specifically, findings such as those of Nigro and coworkers (2018) confirmed that when *B. subtilis* strain QST 713 strain (Serenade Max^®^) was applied on a monthly basis between April and October, it appeared to be equally effective as chemical fungicides in reducing both the incidence of infections and the severity of the disease in olive trees [15]. However, it failed to maintain these outcomes in December, possibly due to the repeated rainfall that occurred in Southern Italy, where the experimental olive groves were located. In the framework of this study, from the investigation of the expression levels of ten defense genes post application of biological Serenade^®^ ASO and Sonata^®^, four genes demonstrated increased levels of expression compared to the mock-treated plants, which refers to plants that had not undergone any intervention. The lipoxygenase gene *Lox* was found to be upregulated post the application of both plant protection products, while, on the contrary, the expression of *Pal* and *Bglu* was induced post the application of Serenade^®^ ASO. Sonata^®^-treated plants that were subsequently artificially inoculated with *C. acutatum* displayed relatively elevated levels of the *Lox* gene in comparison to the plants treated only with this bioPPP. Lipoxygenases are enzymes that catalyze the production of oxylipins, which are signaling molecules involved in the immune responses of plants against plant pathogens [51]. Accordingly, the *Pal* gene is the core enzyme for the phenylpropanoid pathway, as it plays a crucial role in the formation of phenolic compounds and the biosynthesis of salicylic acid (SA). Finally, *BGLU* is involved in the degradation of phenolic compounds and, thus, plays an important role in the formation of lignin and lignin derivatives, compounds that have been shown to possess antimicrobial properties [52,53]. As indicated above, biological plant protection products containing *Bacillus* bacteria can be an effective solution for controlling olive anthracnose. Their use is mainly preventive, as their action is focused on the early developmental stages of the fungus [26]. Due to its existing approval, Serenade^®^ ASO could be part of an integrated disease management program (IPM) and used in combination with the registered chemical fungicides applied to control anthracnose, especially during autumn, when the use of synthetic fungicides is not recommended due to their residues on the olive drupes and olive oil.

The bioPPP Botector^®^, which contains the yeast *Aureobasidium pullulans*, demonstrated low percentages in reducing disease severity and inhibiting spore production in both olive varieties. Similar experiments were conducted for the control of *C. acutatum* on apple fruits, where *A. pullulans* also demonstrated low inhibitory activity against the growth of the plant pathogenic fungus [54,55]. On the other hand, endophytic strains of *A. pullulans* were applied during the flowering stage, resulting in a reduction in the percentage of latent infections, compared to the control of the experiment [15]. In conclusion, the timing and frequency of application seem to play a crucial role, as the most effective strategy for optimizing the effectiveness of biocontrol agents is their application during the flowering stage, or before harvest to prevent and reduce latent infections [56,57].

The plant protection product Polyversum^®^, containing the biocontrol agent *Pythium oligandrum* strain M1, resulted in low effectiveness in reducing disease severity, with reduction rates ranging from 20–30% for the Koroneiki and Kalamon varieties, respectively. On the other hand, the application of this product showed a decrease in the number of conidia produced by the fungus *C. acutatum*, with inhibition rates exceeding 30% for drupes of *cv.* Koroneiki and 40% for drupes of *cv.* Kalamon. Strains of *Pythium oligandrum* exhibit properties such as competition for space, nutrients, and parasitism of plant pathogenic fungi. Additionally, one of the well-studied mechanisms of action of *P. oligandrum* is its ability to induce plant defense mechanisms through substances it produces, such as oligandrin [58,59]. However, there are no published results investigating the competitive ability of *P. oligandrum* against *Colletotrichum* fungi.

The commercial products Fytosave^®^, LBG 01F34^®^, and Vacciplant^®^ are being classified as biostimulants, as they have the property, due to their composition, to activate the natural defense mechanisms of plants. The active substances that have the ability to induce these defense mechanisms are chito-oligosaccharides (COS-OGA) for Fytosave^®^, potassium phosphonates for LBG 01F34^®^, and laminarin for Vacciplant^®^. Fytosave^®^ did not show any reduction in disease severity or the number of conidia produced by the fungus *C. acutatum* in the detached fruits of *cv.* Koroneiki. On the contrary, on drupes of *cv.* Kalamon, its application led to a reduction in the disease by more than 30% and an above 40% decrease in conidia production compared to the control of the experiment. Comparably, LBG 01F34^®^ and Vacciplant^®^ did not prevent the development of the disease on *cv.* Koroneiki fruit. As for the fruits of *cv.* Kalamon, the two aforementioned products resulted in a reduction in disease severity by approximately 30%. More specifically, LBG 01F34^®^ led to a reduction in conidia production in rates above 40%, while Vacciplant^®^ did not significantly affect spore production by the pathogen. Due to their mechanism of action, the timing of application of biostimulants plays a crucial role in their effectiveness. Their use is preventive, and they are recommended to be used in combination with other management tools. In order to draw further conclusions about their action, monitoring of the expression levels of plant defense genes post the application of Fytosave^®^, LBG 01F34^®^, and Vacciplant^®^ are necessary. The study by Tziros and coauthors (2020) provided encouraging results regarding the induction of defense gene expression in olive plants post the application of the active substance laminarin (Vacciplant^®^) and, consequently, the management of the disease caused by the fungus *Spilocaea oleaginea* [47].

Mevalone^®^ is a registered biological plant protection product in Greece for the control of olive anthracnose, consisting of the active compounds thymol, eugenol, and geraniol. Although it was found to be ineffective in reducing disease severity, it demonstrated a strong inhibition against the production of conidia by *C. acutatum*, leading to a reduction of 60% compared to the control. Analogous results were obtained for *cv.* Kalamon, where Mevalone^®^ did not significantly reduce the symptoms, and the inhibition of conidia production by the pathogen did not exceed 30%. Regarding the investigation of the expression levels of ten selected genes in the presence of Mevalone^®^, *Mpol* and *PR10* displayed an upregulation when compared to the mock-treated plants. The biosynthesis of thymol and its isomer carvacrol is believed to involve the hydroxylation of precursor compounds, such as gamma-terpinene and p-cymene. These compounds are characterized as bioactive, having antimicrobial properties among other qualities [60]. Geraniol and thymol showed high activity against *C. acutatum*, compared to eugenol, as concluded by experiments conducted by Scariot and colleagues (2020) [61]. However, the effectiveness of geraniol and thymol in inhibiting the fungal growth was found to depend on the concentration applied. Furthermore, flow cytometry data showed that thymol, carvacrol, and geraniol can affect the permeability of the fungal cell membrane, reduce its metabolism, and hinder the function of proteins, specifically efflux pumps involved in the release of toxic compounds from pathogenic cells into the external environment [61,62]. The combination of high antifungal activity and low biotransformation level by microorganisms makes thymol and carvacrol promising compounds for controlling *C. acutatum* [63].

## 4. Materials and Methods

### 4.1. Plant Protection Products

A total of 11 biological plant protection products (bioPPPs) classified as commercial were used (Table 1).

### 4.2. In Situ Experiments on Olive Fruits

The detached fruit used for the in situ experiments belonged to the two most important Greek varieties *cv.* Kalamon and *cv.* Koroneiki, for the production of table olives and olive oil, respectively. The drupes were phenotypically noninfected by the fungus and obtained from olive groves where no chemical control was taking place. Two experiments were conducted to evaluate the effectiveness of the 11 commercial products in reducing disease severity on artificially infected fruit of *cv.* Koroneiki and *cv.* Kalamon with *C. acutatum*. Fruits were surface-sterilized in a 10% solution of commercial bleach (13.75% *w*/*w* of sodium hypochlorite-NaOCl) for 10 min, rinsed with sterile water, immersed in 70% ethanol solution (C_2_H_5_OH) for 3 min, rinsed with sterile water, and finally air-dried. Sterilized fruits were immersed in a solution of the plant protection product at different active ingredient doses according to the manufacturer and the maximum applied dose as indicated from the Greek Ministry of Rural Development and Food (2021) (Table 1). Twenty-four hours post fruit treatments, drupes were sprayed with conidial suspension (10^5^ conidia mL^−1^) of the reference *C. acutatum* isolate O9 [5]. The fruits were then placed in 20 × 10 × 8 cm^3^ sterilized plastic containers and incubated in chambers for approximately a month under high-humidity conditions, 25 °C, and switching of light/dark (10/14 h). In all the experiments, untreated inoculated and untreated noninoculated fruits were used as a control to quantify the percentage of latent infection, respectively. A completely randomized design with three replicated humidity chambers per treatment and eight fruits per replicated chamber was used. Disease severity was assessed every 2–3 days by using a 0-to-5 rating scale (Table 2). The disease severity index (DSI) was calculated for each replication using the following formula: DSI = [(Σni × i)/ (N × 6)] × 100, where i represents the severity (0–5), ni is the number of fruits with the severity i, and N is the total number of fruits [14]. The area under the disease progress curve (AUDPC) was calculated by the trapezoidal integration of DI values over time. The total number of conidia produced for each treatment was counted via microscopy.

### 4.3. Defense Related Genes Expression

The expression levels of 10 genes (Table 3) known to be involved in the olive defense mechanisms were analyzed [47,64,65,66,67] using the RT-qPCR method in plants treated with the four most effective biological plant protection products (Mevalone^®^ Sonata^®^, Serenade^®^ ASO, Remedier^®^, and Trianum-P^®^) based on these studies. The experiments were carried out on two-year-old plants of *cv.* Kalamon. These plants originated from pathogen-free olive cuttings grown in a commercial olive nursery. Olive trees were grown in individual pots containing soil and kept at 25 ± 3 °C and 50–60% RH. The application of the bioPPPs was carried out 48 h before the artificial inoculation with the *C. acutatum*, and sampling took place 24 h after the inoculation with the pathogen. Immediately after its detachment from the olive trees, the collected leaf material (10 leaves/plant) was immersed in liquid nitrogen and stored at 80 °C until used for further analysis. For each treatment, three replicated plants were used. A multitreatment experiment was conducted to determine the relative gene expression patterns in (a) untreated plants artificially inoculated with *C. acutatum* (positive control), (b) bioPPP-treated and artificially inoculated plants, (c) bioPPP-treated plants, and (d) mock-treated plants (negative control). For the artificial inoculation, conidial suspension of 10^5^ conidia mL^−1^ was prepared from pure PDA cultures of the *C. acutatum* strain O9 which had previously been grown for seven days under a light/dark cycle (10/14 h) at 25 °C. A 0.05% Tween solution was added to the suspension. An equal amount of inoculum was applied to all treatments through spraying, except for the negative control and the bioPPP-treated plants, which were sprayed with an equivalent amount of water. For each treatment, three biological replicates and three technical replicates were composed. The collected leaves were ground to a fine powder using liquid nitrogen and stored at −80 °C until use. Subsequently, total RNA was extracted from 250 mg of tissue using the Nucleo Spin RNA Plant kit (Macherey-Nagel, GmbH & Co. KG, Düren, Germany) according to the manufacturer’ s instructions. The total RNA extracted was used as a template for RT-qPCR, and the cDNA was created using FIREScript RT cDNA Synthesis KIT, SolisBioDyne according to the manufacturer’s instructions. The RT-qPCR reactions were performed using a StepOne Plus Real-Time PCR System (Applied Biosystems, Waltham, MA, USA) using an SYBR Green based kit (KAPA SYBR^®^ FAST qPCR, Master Mix (2X) Kit, KAPA BIOSYSTEMS, Wilmington, MA, USA) according to the manufacturer’s instructions and the primers indicated in Table 3. The amplification conditions were 55 °C for 10 min, 95 °C for 2 min, followed by 40 cycles of 95 °C for 10 s and 60 °C for 1 min, while the melt curve stage consisted of 95 °C for 15 s, 60 °C for 1 min, and 95 °C for 15 s [47]. The threshold cycle (Ct) was determined using the default threshold settings. The 2^−∆∆Ct^ method was applied to calculate the relative gene expression levels. The actin gene was used as the endogenous control. For the multitreatment gene expression experiment, samples were normalized with the untreated/mock-inoculated plants.

## 5. Conclusions

Overall, the products that reduced disease severity or effectively inhibited conidia production by *Colletotrichum acutatum* were Remedier^®^, Trianum-P^®^, Serenade^®^ ASO, Sonata^®^, and Mevalone^®^. Moreover, in the aforementioned biological control products-treated plants, several olive defense genes showcased overexpression, as compared to the control of the experiment. For the development and application of specific bioPPPs, certain modes of action are preferred in order to prevent possible resistance development. Thus, the findings of this study highlight the potential of biological plant protection products (bioPPPs) to serve as effective tools for the control of olive anthracnose within an integrated pest management (IPM) framework. More experimentation needs to be carried out under field conditions, also taking into account the timing of bioPPPs application. By considering the specific properties and modes of action of the commercial biological plant protection products tested in the current study, olive producers will be able to optimize their pest management practices, leading to more sustainable and resilient olive production systems.

## Figures and Tables

**Table 1:** List of the biological plant protection products (bioPPPs).

Biological Plant Protection Products	Active Substances	Maximum Certified Dose	Supplier
Amylo-X^®^ WG	*Bacillus amyloliquefaciens* subsp. *plantarum* strain D747	1.5 g L^−1^	K&N Efthymiadis S.A., Athens, Greece
Botector^®^ WG	*Aureobasidium pullulans* DSM 14940; *Aureobasidium pullulans* DSM 14941	1 g L^−1^	Elanco Hellas S.A., Avlonas, Greece
FytoSave^®^ SL	Chito-oligosaccharides (COS-OGA)	3.3 mL L^−1^	Elton International Trading Co. S.A., Athens, Greece
LBG 01F34^®^ SL	Potassium phosphonates	4 mL L^−1^	BASF Hellas S.A., Athens, Greece
Mevalone^®^ CS	Eugenol, Thymol, Geraniol	4 mL L^−1^	K&N Efthymiadis S.A., Athens, Greece
Polyversum^®^ WP	*Pythium oligandrum* strain M1	0.3 g L^−1^	BASF Hellas S.A., Athens, Greece
Remedier^®^ WP	*Trichoderma asperellum* strain ICC012 *Trichoderma gamsii* strain ICC080	2.5 g L^−1^	AGROLOGY S.A., Athens, Greece
Serenade Aso^®^ SC	*Bacillus amyloliquefaciens* strain QST 713	10 mL L^−1^	Bayer Hellas A.G., Athens, Greece
Sonata^®^ SC	*Bacillus pumilus* strain QST 2808	1 mL L^−1^	Bayer Hellas A.G., Athens, Greece
Trianum-P^®^ WG	*Trichoderma harzianum* strain T22	0.3 g L^−1^	Koppert B.V. Hellas, Athens, Greece
Vacciplant^®^ SL	Laminarin	2 mL L^−1^	Alfa Agricultural Supplies S.A., Athens, Greece

**Table 2:** Disease severity assessment scale [14].

Number	Percentage of Fruit Rot
0	No visible symptoms
1	Visible symptoms affecting less than 25% of the fruit surface
2	Visible symptoms affecting 25–50% of the fruit surface
3	Visible symptoms affecting 50–75% of the fruit surface
4	Visible symptoms affecting 75–100% of the fruit surface
5	Fruit completely rotten

**Table 3:** Primer sequences used for gene expression analysis in RT-qPCR assays.

Gene	Primers	Primer Sequence (5′–3′)	Product Size (bp)	Accession Number	Reference
Phenylalanine ammonia-lyase (*Pal*)	OePAL-F	CATTGAAAGGTAGCCATCTA	101	KJ511868	[65]
	OePAL-R	CTAGCAAATTGGAAGAGGTT			
Copper amin oxidase (*Cuao*)	OeCUAO-F	AAGATGGCCTTGGGAAGAAT	191	GQ851613	[47]
	OeCUAO-R	TTCTGCCAATCCTGTTCTCC			
Putative alcohol dehydrogenase (*Aldh1*)	OeALDH1-F	TTTAAGTGGGGAGCTCAAATACA	200	JX266197	
	OeALDH1-R	GATGCTTCAGATATTCCCATGC			
Beta-1,3-glucanase (*Bglu*)	BGLU-F	TTTCACGCGTTGGTAATCCG	180	AJ810085.1	
	BGLU-R	CAGCCTTTTCAAGTGCTGCA			
Major pollen allergen (*Mpol*)	Mpol-F	TGTTCCCCAACCTCCAGTTT	186	XM_0230363	
	Mpol-R	TCCTTCTGCTCTCGTGTAACC			
9-Lipoxygenase (*Lox*)	LOX-F	CAAGCGAAACACCAGAACCA	180	EU678670.1	
	LOX-R	CCACGGATCCTCCAAGAACC			
Phenylalanine ammonia-lyase (*Phely*)	OlPhely-F	CAAAAGCCTAAACAAGATCG	188	XM_023030332.1	
	OlPhely-R	CAGGGGTGGCTTGAAAATTC			
Chitinase 2 (*CHI 2*)	Olest73-F	ACGCTAGTGCAGCAAGTATGACAAGGAGA	145	CK087221	
	Olest73-R	GGAGCCGTGGCGGTCCCACT			
Pathogenesis-related Protein (*PR10*)	PR10-F	GATGTGTGGAGAGGCTTT	153	JZ823324	[64]
	PR10-R	CGTCATTTTTCTTCCTAGG			
Thaumatine-like protein (*PR5*)	PR5-F	GGGCAAGTGAACAGGCTT	177	JZ844402.1	[66]
	PR5-R	GGCGGTTGTAACAACCCGT			
Actin	OlActin-F	GAGCGGGAAATTGTGAGAGA	195	AF545569	[47]
	OlActin-R	CTGGTAAAGAACCTCAGGAC			

## Data Availability

The datasets generated during and/or analyzed during the current study are available from the corresponding author on reasonable request. The data are not publicly available due to potential further exploitation actions.

## References

[1] Moral J., De Oliveira R., Trapero A. (2008). Elucidation of the Disease Cycle of Olive Anthracnose Caused by *Colletotrichum acutatum*. Phytopathology.

[2] Cacciola S.O., Faedda R., Sinatra F., Agosteo G.E., Schena L., Frisullo S., Magnano di San Lio G. (2012). Olive Anthracnose. J. Plant Pathol..

[3] Licciardello G., Moral J., Strano M.C., Caruso P., Sciara M., Bella P., Sorrentino G., Di Silvestro S. (2022). Characterization of *Colletotrichum* Strains Associated with Olive Anthracnose in Sicily. Phytopathol. Mediterr..

[4] Talhinhas P., Mota-Capitão C., Martins S., Ramos A.P., Neves-Martins J., Guerra-Guimarães L., Várzea V., Silva M.C., Sreenivasaprasad S., Oliveira H. (2011). Epidemiology, Histopathology and Aetiology of Olive Anthracnose Caused by *Colletotrichum acutatum* and *C. gloeosporioides* in Portugal. Plant Pathol..

[5] Iliadi M.K., Tjamos E.C., Antoniou P.P., Tsitsigiannis D.I. (2017). First Report of *Colletotrichum acutatum* Causing Anthracnose on Olives in Greece. Plant Dis..

[6] Sergeeva V., Nair N.G., Spooner-Hart R. (2008). Evidence of Early Flower Infection in Olives (*Olea europaea*) by *Colletotrichum acutatum* and *C. gloeosporioides* Causing Anthracnose Disease. Australas. Plant Dis. Notes.

[7] Moreira K.A., Oliveira J.T.C., da Silva E.G., da Rocha A.T., de Medeiros E.V., de Carvalho J.S.B., de Lima J.R.S. (2021). Resistance Induction Anthracnose Control in Pepper Plants Using Acibenzolar-S-Methyl. Divers. J..

[8] Azevedo-Nogueira F., Martins-Lopes P., Gomes S. (2020). Current Understanding of *Olea europaea* L.—*Colletotrichum acutatum* Interactions in the Context of Identification and Quantification Methods—A Review. Crop Prot..

[9] Moreira V., Ferronato B., de Benedetti F., González-Barrios P., Mondino P., Alaniz S. (2022). Incidence of *Colletotrichum* Latent Infections during Olive Fruit Development under Uruguayan Environmental Conditions. Int. J. Pest Manag..

[10] Schena L., Mosca S., Cacciola S.O., Faedda R., Sanzani S.M., Agosteo G.E., Sergeeva V., Magnano di San Lio G. (2014). Species of the *Colletotrichum gloeosporioides* and *C. boninense* Complexes Associated with Olive Anthracnose. Plant Pathol..

[11] Talhinhas P., Loureiro A., Oliveira H. (2018). Olive Anthracnose: A Yield- and Oil Quality-Degrading Disease Caused by Several Species of *Colletotrichum* That Differ in Virulence, Host Preference and Geographical Distribution. Mol. Plant Pathol..

[12] Kolainis S., Koletti A., Lykogianni M., Karamanou D., Gkizi D., Tjamos S.E., Paraskeuopoulos A., Aliferis K.A. (2020). An Integrated Approach to Improve Plant Protection against Olive Anthracnose Caused by the *Colletotrichum acutatum* Species Complex. PLoS ONE.

[13] Martins F., Pereira J.A., Baptista P. (2019). Olive Anthracnose and Its Management by Fungal Endophytes: An Overview. Plant Microbe Interface.

[14] Moral J., Agustí-Brisach C., Agalliu G., de Oliveira R., Pérez-Rodríguez M., Roca L.F., Romero J., Trapero A. (2018). Preliminary Selection and Evaluation of Fungicides and Natural Compounds to Control Olive Anthracnose Caused by *Colletotrichum* Species. Crop Prot..

[15] Nigro F., Antelmi I., Sion V., Pacifico A. (2019). Integrated Approaches to Control Fungi Affecting the Canopy of Olive Trees. IOBC-WPRS Bull..

[16] Materatski P., Varanda C., Carvalho T., Campos M.D., Gomes L., Nobre T., Rei F. (2019). Plants Virulence and Diversity of *Colletotrichum* spp. Associated to Olive Anthracnose. Plants.

[17] Sanders G.M., Korsten L. (1999). Survey of Fungicide Resistance of *Colletotrichum gloeosporioides* from Different Avocado Production Areas. S. Afr. Avocado Grow. Assoc. Yearb..

[18] Busby P.E., Ridout M., Newcombe G. (2016). Fungal Endophytes: Modifiers of Plant Disease. Plant Mol. Biol..

[19] Gao F.K., Dai C.C., Liu X.Z. (2010). Mechanisms of Fungal Endophytes in Plant Protection against Pathogens. Afr. J. Microbiol. Res..

[20] Speckbacher V., Zeilinger S. (2018). Secondary Metabolites of Mycoparasitic Fungi. Second. Metab. Appl..

[21] Poveda J., Baptista P. (2021). Filamentous Fungi as Biocontrol Agents in Olive (*Olea Europaea* L.) Diseases: Mycorrhizal and Endophytic Fungi. Crop Prot..

[22] Lorito M., Woo S.L., Lugtenberg B. (2015). Trichoderma: A Multi-Purpose Tool for Integrated Pest Management BT. Principles of Plant-Microbe Interactions: Microbes for Sustainable Agriculture.

[23] Moreira R.R., Nesi C.N., May De Mio L.L. (2014). *Bacillus* spp. and *Pseudomonas putida* as Inhibitors of the *Colletotrichum acutatum* Group and Potential to Control Glomerella Leaf Spot. Biol. Control.

[24] Trotel-Aziz P., Couderchet M., Biagianti S., Aziz A. (2008). Characterization of New Bacterial Biocontrol Agents Acinetobacter, *Bacillus*, *Pantoea* and *Pseudomonas* spp. Mediating Grapevine Resistance against *Botrytis cinerea*. Environ. Exp. Bot..

[25] Punja Z.K., Utkhede R.S. (2003). Using Fungi and Yeasts to Manage Vegetable Crop Diseases. Trends Biotechnol..

[26] Kupper K.C., Corrêa F.E., de Azevedo F.A., da Silva A.C. (2012). *Bacillus subtilis* to Biological Control of Postbloom Fruit Drop Caused by *Colletotrichum acutatum* under Field Conditions. Sci. Hortic..

[27] Klein M.N., da Silva A.C., Kupper K.C. (2016). *Bacillus subtilis* Based-Formulation for the Control of Postbloom Fruit Drop of Citrus. World J. Microbiol. Biotechnol..

[28] Yenjit P., Intanoo W., Chamswarng C., Siripanich J., Intana W. (2004). Use of Promising Bacterial Strains for Controlling Anthracnose on Leaf and Fruit of Mango Caused by *Colletotrichum gloeosporioides*. Walailak J. Sci. Technol..

[29] Sahile S., Fininsa C., Sakhula P.K., Ahmed S. (2009). Evaluation of Pathogenic Isolates in Ethiopia for the Control of Chocolate Spot in Faba Bean. Afr. Crop Sci. J..

[30] Lamsal K., Kim S.W., Kim Y.S., Lee Y.S. (2012). Application of Rhizobacteria for Plant Growth Promotion Effect and Biocontrol of Anthracnose Caused by *Colletotrichum acutatum* on Pepper. Mycobiology.

[31] Pangallo S., Li Destri Nicosia M.G., Agosteo G.E., Abdelfattah A., Romeo F.V., Cacciola S.O., Rapisarda P., Schena L. (2017). Evaluation of a Pomegranate Peel Extract as an Alternative Means to Control Olive Anthracnose. Phytopathology.

[32] Thomashow L.S. (1996). Biological Control of Plant Root Pathogens. Curr. Opin. Biotechnol..

[33] Durrant W.E., Dong X. (2004). Systemic Acquired Resistance. Annu. Rev. Phytopathol..

[34] Kloepper J.W., Ryu C.-M., Zhang S. (2004). Induced Systemic Resistance and Promotion of Plant Growth by *Bacillus* spp.. Phytopathology.

[35] Chowdhury S.P., Hartmann A., Gao X., Borriss R. (2015). Biocontrol Mechanism by Root-Associated *Bacillus amyloliquefaciens* FZB42—A Review. Front. Microbiol..

[36] Kamle M., Borah R., Bora H., Jaiswal A.K., Singh R.K., Kumar P., Hesham A.E.-L., Upadhyay R.S., Sharma G.D., Manoharachary C., Gupta V.K. (2020). Systemic Acquired Resistance (SAR) and Induced Systemic Resistance (ISR): Role and Mechanism of Action Against Phytopathogens BT—Fungal Biotechnology and Bioengineering.

[37] Freeman S., Barbul O., David D.R., Nitzani Y., Zveibil A., Elad Y. (2001). *Trichoderma* spp. for Biocontrol of *Colletotrichum acutatum* and *Botrytis cinerea* in Strawberry. IOBC WPRS Bull..

[38] Freeman S., Minz D., Kolesnik I., Barbul O., Zveibil A., Maymon M., Nitzani Y., Kirshner B., Rav-David D., Bilu A. (2004). *Trichoderma* Biocontrol of *Colletotrichum acutatum* and *Botrytis cinerea* and Survival in Strawberry. Eur. J. Plant Pathol..

[39] Es-Soufi R., El Bouzdoudi B., Bouras M., El Kbiach M.L., Badoc A., Lamarti A. (2017). Assessment of the Effect of Environmental Factors on the Antagonism of *Bacillus amyloliquefaciens* and *Trichoderma harzianum* to *Colletotrichum acutatum*. Adv. Microbiol..

[40] Es-Soufi R., Tahiri H., Azaroual L., El Oualkadi A., Martin P., Badoc A., Lamarti A. (2020). In Vitro Antagonistic Activity of *Trichoderma harzianum* and *Bacillus amyloliquefaciens* against *Colletotrichum acutatum*. Adv. Microbiol..

[41] Dennis C., Webster J. (1971). Antagonistic Properties of Species-Groups of *Trichoderma*: II. Production of Volatile Antibiotics. Trans. Br. Mycol. Soc..

[42] Živković S., Stojanović S., Ivanović Ž., Gavrilović V., Popović T., Balaž J. (2010). Screening of antagonistic activity of microorganisms against *Colletotrichum acutatum* and *Colletotrichum gloeosporioides*. Arch. Biol. Sci..

[43] Van Loon L.C., Rep M., Pieterse C.M.J. (2006). Significance of Inducible Defense-Related Proteins in Infected Plants. Annu. Rev. Phytopathol..

[44] Bufe A., Spangfort M.D., Kahlert H., Schlaak M., Becker W.-M. (1996). The Major Birch Pollen Allergen, Bet v 1, Shows Ribonuclease. Act. Planta.

[45] Van Loon L.C., Van Strien E.A. (1999). The Families of Pathogenesis-Related Proteins, Their Activities, and Comparative Analysis of PR-1 proteins. Physiol. Mol. Plant Pathol..

[46] Rockenbach M.F., Velho A.C., Alaniz S.M., Stadnik M.J. (2018). Resistance of Apple Leaves to Infection by *Colletotrichum fructicola* Acts Independently of Hypersensitive Reaction and *PR-1* and *PR-10* Gene Expression. Trop. Plant Pathol..

[47] Tziros G.T., Samaras A., Karaoglanidis G.S. (2021). Laminarin Induces Defense Responses and Efficiently Controls Olive Leaf Spot Disease in Olive. Molecules.

[48] Swinburne T.R., Barr J.G., Brown A.E. (1975). Production of Antibiotics by *Bacillus subtilis* and Their Effect on Fungal Colonists of Apple Leaf Scars. Trans. Br. Mycol. Soc..

[49] Serrano L., Manker D., Brandi F., Cali T. The Use of *Bacillus subtilis* QST 713 and *Bacillus pumilus* QST 2808 as Protectant Fungicides in Conventional Application Programs for Black Leaf Streak Control. Proceedings of the VII International Symposium on Banana: ISHS-ProMusa Symposium on Bananas and Plantains: Towards Sustainable Global Production 986.

[50] Bizos G., Papatheodorou E.M., Chatzistathis T., Ntalli N., Aschonitis V.G., Monokrousos N. (2020). The Role of Microbial Inoculants on Plant Protection, Growth Stimulation, and Crop Productivity of the Olive Tree (*Olea Europea* L.). Plants.

[51] Marcos R., Izquierdo Y., Vellosillo T., Kulasekaran S., Cascón T., Hamberg M., Castresana C. (2015). 9-Lipoxygenase-Derived Oxylipins Activate Brassinosteroid Signaling to Promote Cell Wall-Based Defense and Limit Pathogen Infection. Plant Physiol..

[52] Medina E., De Castro A., Romero C., Brenes M. (2006). Comparison of the Concentrations of Phenolic Compounds in Olive Oils and Other Plant Oils: Correlation with Antimicrobial Activity. J. Agric. Food Chem..

[53] Omar S.H. (2010). Oleuropein in Olive and Its Pharmacological Effects. Sci. Pharm..

[54] Di Francesco A., Ugolini L., Lazzeri L., Mari M. (2015). Production of Volatile Organic Compounds by *Aureobasidium pullulans* as a Potential Mechanism of Action against Postharvest Fruit Pathogens. Biol. Control.

[55] Mari M., Martini C., Spadoni A., Rouissi W., Bertolini P. (2012). Biocontrol of Apple Postharvest Decay by *Aureobasidium pullulans*. Postharvest Biol. Technol..

[56] Ippolito A., Nigro F. (2000). Impact of Preharvest Application of Biological Control Agents on Postharvest Diseases of Fresh Fruits and Vegetables. Crop Prot..

[57] Schena L., Nigro F., Pentimone I., Ligorio A., Ippolito A. (2003). Control of Postharvest Rots of Sweet Cherries and Table Grapes with Endophytic Isolates of *Aureobasidium pullulans*. Postharvest Biol. Technol..

[58] Yacoub A., Gerbore J., Magnin N., Chambon P., Dufour M.C., Corio-Costet M.F., Guyoneaud R., Rey P. (2016). Ability of *Pythium oligandrum* Strains to Protect *Vitis vinifera* L., by Inducing Plant Resistance against *Phaeomoniella chlamydospora*, a Pathogen Involved in Esca, a Grapevine Trunk Disease. Biol. Control.

[59] Mohamed N., Lherminier J., Farmer M.-J., Fromentin J., Béno N., Houot V., Milat M.-L., Blein J.-P. (2007). Defense Responses in Grapevine Leaves Against *Botrytis cinerea* Induced by Application of a *Pythium oligandrum* Strain or Its Elicitin, Oligandrin, to Roots. Phytopathology.

[60] Naghdi Badi H., Abdollahi M., Mehrafarin A., Ghorbanpour M., Tolyat M., Qaderi A., Ghiaci Yekta M. (2017). An Overview on Two Valuable Natural and Bioactive Compounds, Thymol and Carvacrol, in Medicinal Plants. J. Med. Plants.

[61] Scariot F.J., Foresti L., Delamare A.P.L., Echeverrigaray A.P.L.S. (2020). Activity of Monoterpenoids on the in Vitro Growth of Two *Colletotrichum* Species and the Mode of Action on *C. acutatum*. Pestic. Biochem. Physiol..

[62] Nazzaro F., Fratianni F., Coppola R., Feo V. (2017). De Essential Oils and Antifungal Activity. Pharmaceuticals.

[63] Oviedo L.A., García C.M., Durango D.L., Numpaque M.A., Gil J.H. (2011). Thymol and Carvacrol: Biotransformation and Antifungal Activity against the Plant Pathogenic Fungi *Colletotrichum acutatum* and *Botryodiplodia theobromae*. Trop. Plant Pathol..

[64] Cabanás C.G.L., Schilirò E., Valverde-Corredor A., Mercado-Blanco J. (2015). Systemic Responses in a Tolerant Olive (*Olea europaea* L.) Cultivar upon Root Colonization by the Vascular Pathogen *Verticillium dahliae*. Front. Microbiol..

[65] Trabelsi R., Sellami H., Gharbi Y., Cheffi M., Chaari A., Baucher M., El Jaziri M., Triki M.A., Gdoura R. (2017). Response of Olive Tree (*Olea europaea* L. cv. Chemlali) to Infection with Soilborne Fungi. J. Plant Dis. Prot..

[66] Ben Amira M., Lopez D., Triki Mohamed A., Khouaja A., Chaar H., Fumanal B., Gousset-Dupont A., Bonhomme L., Label P., Goupil P. (2017). Beneficial Effect of *Trichoderma harzianum* Strain Ths97 in Biocontrolling *Fusarium solani* Causal Agent of Root Rot Disease in Olive Trees. Biol. Control.

[67] Gouvinhas I., Martins-Lopes P., Carvalho T., Barros A., Gomes S. (2019). Impact of *Colletotrichum acutatum* Pathogen on Olive Phenylpropanoid Metabolism. Agriculture.

